# Emerging liquid biopsy markers in thyroid cancer: from isolation technologies to clinical translation

**DOI:** 10.3389/fendo.2026.1757547

**Published:** 2026-03-31

**Authors:** Yujia Gao, Qiyue Duan, Yufei Wei, Xiaolin Hou, Hongxia Fu, Shujuan Wang, Ling Li, Huiling Li

**Affiliations:** 1 Department of Medical Laboratory, First Hospital of Shanxi Medical University, Taiyuan, China; 2 Department of Nuclear Medicine, First Hospital of Shanxi Medical University, Taiyuan, China; 3 Collaborative Innovation Center for Molecular Imaging of Precision Medicine, Shanxi Medical University, Taiyuan, China

**Keywords:** circulating tumor cells (CTCs), circulating tumor DNA (ctDNA), exosomes, liquid biopsies (LB), thyroid cancer (TC)

## Abstract

Diagnosis and treatment of thyroid cancer (TC) are undergoing radical changes, shifting from traditional tissue studies to liquid biopsies (LB). LB technologies provide effective, non-invasive solutions to these challenges. This paper reviews recent advances in the analysis of three important biomarker systems: circulating tumor cells (CTCs), circulating tumor DNA (ctDNA) and exosomes. Technically, microfluidics and bioaffinity-based physical capture strategies have significantly improved the efficiency with which CTCs can be separated and analysed at an individual cell level. Ultra-sensitive digital polymerase chain reaction (dPCR) and targeted sequencing enable accurate characterization of rare mutations and methylation alterations in ctDNA. Additionally, innovations in ultracentrifugation and new microfluidic chips have improved the efficiency with which exosomes and the information molecules they contain (e.g., miRNA and lncRNA) are extracted and detected. These abundant liquid biomarkers demonstrate significant value in clinical translation. CTCs provide a unique opportunity to study the mechanisms of metastasis and tumor heterogeneity, while ctDNA plays a central role in preoperative risk classification, monitoring minimal residual disease and tracking the dynamics of drug-resistant mutations. Exosomes, as stable carriers of nucleic acids, have significant potential for early diagnosis and assessment of treatment efficacy. Nevertheless, this field still faces significant challenges, including a lack of technical standardization and integration of different biomarker results, insufficient sensitivity in patients with low tumor burden and a lack of broad preclinical validation. In the future, joint analysis of multigenomic biomarkers and deep integration of the latest technologies, such as microfluidics and artificial intelligence, promise to create a more comprehensive ecosystem for precise TC diagnosis and treatment.

## Introduction

Malignant thyroid tumors are the most common type of cancer in the endocrine system. Over the past three decades, the global incidence rate has been continuously increasing, and the growth rate has accelerated significantly since 2000 (1). According to data from the American Cancer Society, in 2021, there were 44,280 new cases of thyroid cancer (TC) in the United States, with 2,200 deaths (2). As we all known, the well-differentiated subtypes originating from follicular cell mainly include papillary thyroid cancer (PTC), follicular thyroid cancer (FTC) and oncocytic carcinoma of the thyroid (OCA, previously known as Hürthle cell cancer), collectively referred to as differentiated thyroid cancer (DTC) (3). The initial two subtypes characteristically exhibit a favorable prognosis, with PTC attaining a 10-year survival rate that approaches 95% (4, 5). While anaplastic thyroid cancer (ATC) is a rare and highly invasive type with a very poor prognosis (6). Medullary thyroid carcinoma (MTC), ATC, and poorly differentiated thyroid carcinoma (PDTC) are less prevalent but exhibit a higher degree of malignancy (7).

Fine-needle aspiration biopsy (FNAB) is the most commonly used technique for clinical diagnosis of TC, featuring simple operation, few complications, and low cost (8). This method collects cell samples from the lesion site for analysis, which is less invasive than incisional or excisional biopsy (9). However, FNAB may yield “non-diagnostic” or “meaningless” results due to sample quality or atypical cell morphology (10). The 2023 “Bethesda System for Reporting Thyroid Cytopathology” classifies FNAB results into six diagnostic categories: (I) nondiagnostic; (II) benign; (III) atypia of undetermined significance; (IV) follicular neoplasm; (V) suspicious for malignancy; and (VI) malignant (11). It is estimated that 25% of FNAB samples from thyroid nodules are classified as “indeterminate” samples (i.e., Bethesda categories III and IV), and such cases generally necessitate diagnostic test intervention to substantiate a cancer diagnosis (12). Furthermore, FNAB may also cause rare complications, such as local carotid artery wall hematoma and needle tract implantation metastasis reported by Cappelli et al (13).

Although serum thyroglobulin (Tg) testing is recommended for long-term postoperative monitoring to assess the risk of recurrence, its predictive value remains controversial (14). A study of 16 patients showed that 5 cases had no detectable serum Tg after surgery, while the urine exosomal Tg levels were elevated, suggesting a possible missed diagnosis with serum testing (15). Moreover, serum Tg testing is susceptible to interference from thyroglobulin antibodies (TgAb), affecting the interpretation of results (16). Therefore, when employing the ARCHITECT Anti-Tg testing system, the cutoff value is ≤ 1.0 IU/mL (17).

**Figure 1:**
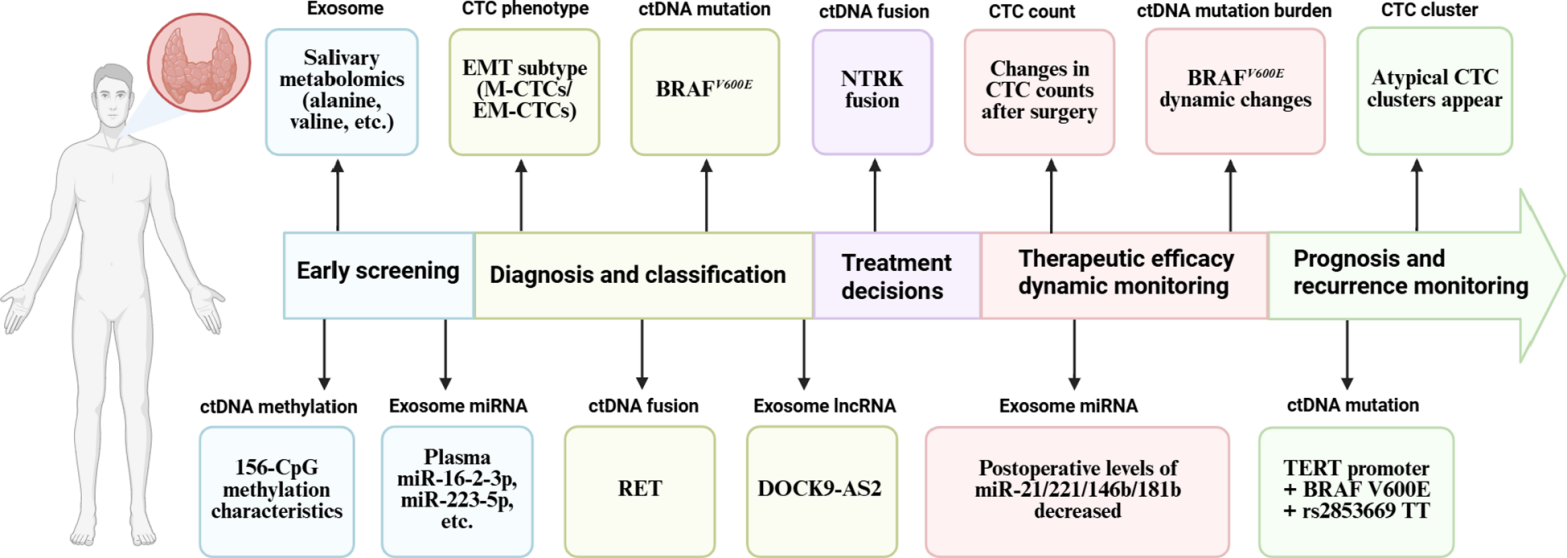
The timeline delineates the temporal progression of the implementation of LB biomarkers throughout the diagnostic and therapeutic continuum of TC. (Created with BioRender.com).

Given the inherent limitations of aforementioned traditional detection methods, the focus of oncology research has gradually shifted from whole-tissue analysis to the detection of tumor-derived substances in body fluids, namely liquid biopsy (LB). This technology enables non-invasive and convenient tumor detection and dynamic monitoring by analyzing tumor-related markers in biological fluids such as blood, urine, and cerebrospinal fluid, etc. (18). As shown in **Figure 1**, we mapped the timeline of LB biomarkers throughout the TC diagnosis and treatment cycle. This demonstrated the potential utility of different biomarkers at key stages, including diagnosis, treatment decision-making, monitoring efficacy, and prognostic follow-up. Compared with traditional methods, LB has several advantages: it can reflect the overall status of the tumor, overcome tumor heterogeneity, has higher diagnostic sensitivity, and supports repeated sampling for real-time tracking of disease progression (19, 20). For instance, a preoperative diagnosis model for TC based on salivary metabolomics achieves high accuracy through the combination of multiple amino acids (area under the receiver operating characteristic curve (AUC) = 0.936, sensitivity 91.2%, specificity 85.2%) (21).

**Figure 2:**
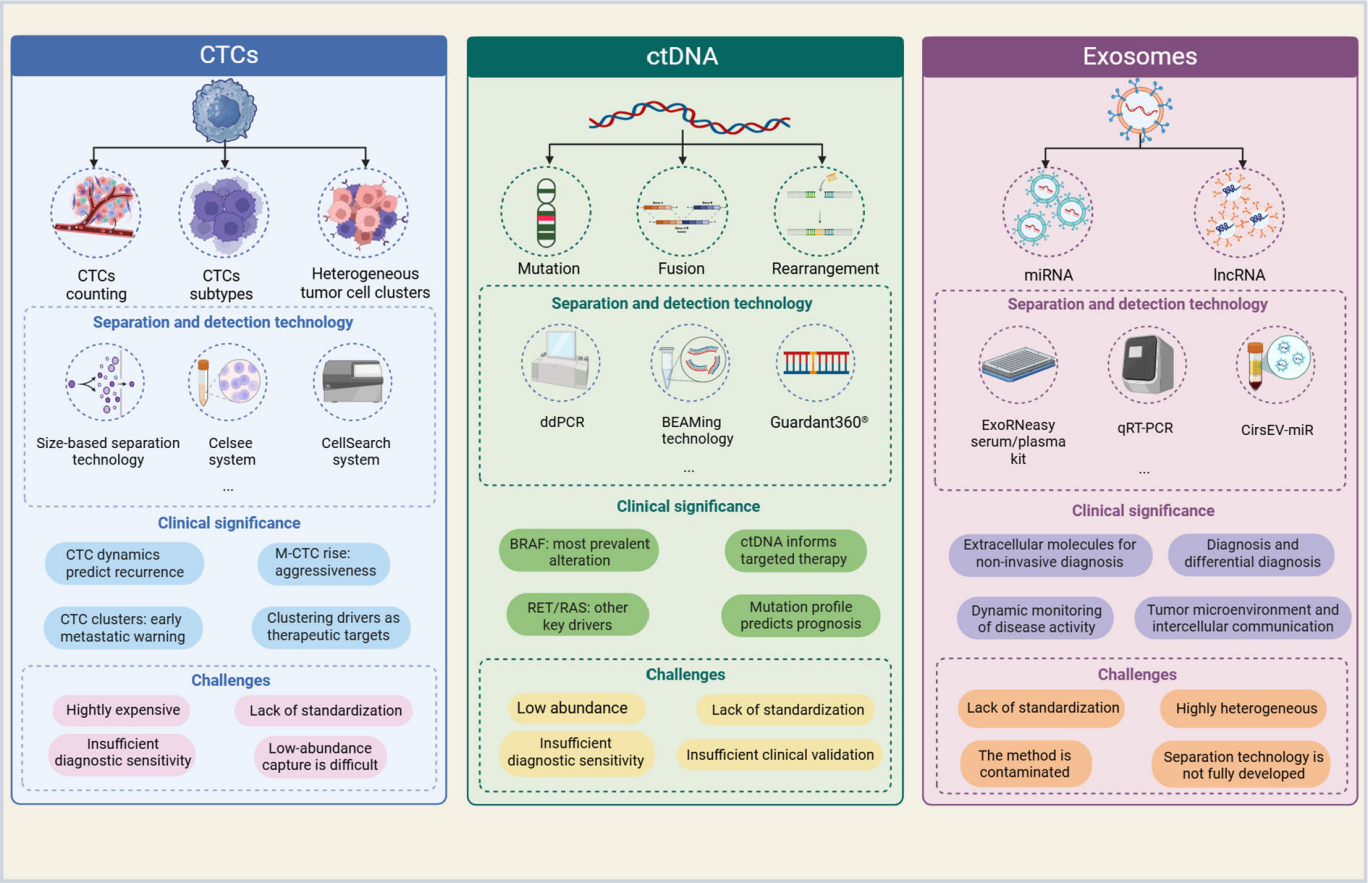
Separation and detection technologies for three major LB markers: the clinical significance and limitations of CTCs, ctDNA and exosome analysis. (Created with BioRender.com).

Currently, the primary cancer biomarkers commonly used in LB technology include circulating tumor cells (CTCs), circulating tumor DNA (ctDNA), and exosomes (as shown in **Figure 2**), which collectively form the core analytical targets of this technology (22). The field of LB is increasingly converging with multi-omics technologies, such as radiomics and artificial intelligence. This convergence significantly improves the ability to detect cancer, assess prognosis and monitor treatment, and is particularly notable in terms of early detection and personalized medicine (23, 24). One of the most critical elements in future development is technological integration and multimodal analysis.

This review aims to provide a comprehensive and critical overview of technological advancements and the current status of the clinical translation of LB biomarkers for TC, with a particular focus on CTCs, ctDNA and exosomes. The latest developments in isolation techniques and detection platforms will be examined, alongside their translational clinical applications in diagnosis, prognostic assessment, and treatment monitoring. This article covers the main types of TC, including DTC, such as PTC, FTC, and OCA, MTC, ATC, whilst addressing both early-stage and advanced/metastatic disease contexts. The article is intended for clinical endocrinologists, translational researchers and laboratory diagnostics specialists, with the aim of bridging the gap between technological advances and clinical practice. It clarifies the value of LB in diagnosing and managing TC and highlights the challenges associated with its practical application.

## Circulating tumor cells

CTCs refer to cancer cells that have detached from primary or metastatic tumor sites and entered the peripheral circulatory system (25). The specific mechanism by which they enter the circulatory system is not yet fully understood, but it may be related to passive shedding during tumor invasive growth or active migration of cancer cells (26, 27). In solid tumors, only approximately 0.0000001% of tumor cells can enter the circulatory system, and the proportion of CTCs in peripheral blood is extremely low, approximately one CTC per million monocytes (27–30). Moreover, the morphological characteristics of CTCs vary depending on the stage and type of tumor (31). They are susceptible to immune clearance, oxidative stress, and mechanical damage, which leads to their scarcity and difficulty in detection in the early stages of cancer (32–34).

Notably, CTCs can be continuously and dynamically monitored, providing an important basis for real-time assessment of changes in tumor biological behavior (35). This characteristic helps to promote timely adjustment of treatment plans and the progress of individualized medicine. Studies have confirmed that compared with traditional blood biomarkers, the dynamic changes in CTC levels have a higher correlation with tumor burden and better diagnostic accuracy (36). Particularly noteworthy is that the reappearance of CTCs after treatment often indicates disease recurrence, highlighting their potential value as an effective treatment monitoring tool (37).

### Separation and detection technology

CTCs separation strategies can be broadly categorized into two main types: one based on physical characteristics (such as size and deformability) and the other based on biological affinity (such as surface markers). Microfluidic technology often serves as a preferred platform for integrating these strategies, as shown in **Table 1**.

**Table 1:** Separation and detection techniques of CTCs used in LB.

Technical classification	Specific technology/platform	Application in TC	Clinical status	Core principle	Key metrics	Ref.
Separation and detection technology based on physical properties	Size-based separation technology	Yes	Applied	Utilize the size difference between CTCs and WBC for sorting.	Sensitivity/recovery rate: low (loss rate approaching 50%)	(38, 39)
	Acoustic amplification sorting technology	No	Under investigation	Using anti-EpCAM/gelatin-coated microspheres to specifically bind CTCs, enhancing acoustic radiation force to achieve separation.	Sensitivity: recovery rate 77%, purity 96%. Specificity: dependent on EpCAM.	(40)
	Celsee system	Yes	Applied	Capture CTCs in microfluidic chip channel networks by exploiting differences in cell size and deformability.	Sensitivity: high-purity CTCs can be obtained. Limitations: small-sized CTCs may be missed.	(42)
Separation technology based on bioaffinity	CellSearch system	Yes	The only CTCs detection platform approved by the FDA.	Immunomagnetic bead enrichment using EpCAM antibodies.	Specificity: strongly EpCAM-dependent.	(43, 44)
	Aptamer-HCR technology	No	Under investigation	Utilizing aptamer recognition to identify CTC surface markers and trigger HCR, forming a DNA hydrogel coating.	Key advantage: enables non-destructive release of CTCs.	(46)
Multifunctional integration platform	Novel microfluidic platform (Size Sorting + Dual-targeted magnetic beads)	No	Applied	Combining size-based sorting (98% recovery rate for spiral chips) with dual-targeted EpCAM/CD146 immunomagnetic beads, and releasing cells via MMP-9 hydrolysis.	Sensitivity: capture efficiency increased by 20%, with high cell viability.	(56)
	Next-generation sequencing (NGS)	Yes	Applied	Sequence CTCs using the Ion Torrent PGM™ system and the Ion AmpliSeq™ Cancer Hotspot Panel.	Key advantage: eliminates bias in whole-genome amplification systems, enhancing the accuracy of mutation detection.	(57)

Size-based separation technology is isolate CTCs from white blood cells (WBCs) based on their difference in diameter (15.6 μm vs. 7-15 μm) (38). For instance, the CanPatrol technology has been employed for the purpose of CTCs separation in TC (39). However, when the size of CTCs is similar to that of WBCs, size exclusion alone can result in the loss of nearly 50% of CTCs (39). Furthermore, fluid turbulence and shear force during the separation process may damage CTCs, thereby affecting the accuracy of subsequent analyses. In order to address the challenge of sorting CTCs, an acoustic amplification sorting technology based on their physical similarity to blood cells was developed (40). This technique employs anti-epithelial cell adhesion molecule (EpCAM)/gelatin-coated microspheres to specifically bind CTCs, thereby enhancing their acoustic radiation force by two orders of magnitude. The efficacy of this method is evidenced by a total recovery rate of 77%, a high purity of 96%, and a high cell viability of 83%, providing highly viable samples for monitoring targeted therapy responses and investigating metastasis mechanisms (40). Hazra et al. (41) developed a novel, label-free, passive microfluidic technique for isolating cancer cells from peripheral blood mononuclear cells within a two-phase aqueous system. Its gradient-decreasing pore size design enables the efficient separation of CTCs from peripheral blood at a processing rate of 25,000 cells per minute with 99% efficiency. The Celsee system utilizes the size and deformability differences of cells to capture CTCs in the capture chambers of its microfluidic chip channel network, while permitting the unimpeded passage of WBCs. This process enables the acquisition of highly purified CTCs (42).

Biological affinity-based separation technologies rely on the binding of ligands, such as antibodies and aptamers, to specific surface antigens on CTCs. The CellSearch technology is currently the only CTCs enrichment platform approved by FDA (43, 44). However, due to its EpCAM-based capture method, it is unable to comprehensively capture cells with diverse phenotypes, which makes it difficult to represent the complete CTC population (43, 44). One study used a “molecular tentacle” chip based on the biotin-streptavidin system, coupling EpCAM antibodies to flexible DNA chains, which expanded the three-dimensional spatial conformation of antibodies and significantly improved the capture efficiency of EpCAM-positive CTCs (45). Song et al. (46) developed a new strategy based on aptamer recognition of CTC surface markers and triggering of a hybridization chain reactions (HCRs). These HCRs formed a DNA hydrogel coating, which enabled the controlled masking and non-destructive release of CTCs. The current mainstream CTCs separation technologies still depend on the binding of surface-specific antigens and ligands (47). Studies have demonstrated that most CTCs express EpCAM on their surface (48). By modifying EpCAM antibodies on the surface of microfluidic separation chips, specific CTCs can be captured (49). Additionally, the *c*lick-*h*andle-loaded M13 *ph*age-based surf*ace* (CHPhace) platform utilizes the low proliferative activity of circulating tumor stem cells in TC to achieve metabolic targeting (50). This platform’s capture capacity is enhanced by multivalent nanofibers, achieving a separation efficiency of over 80%. This technology overcomes the limitations of traditional epithelial cell EpCAM dependence, providing key cellular targets for the non-invasive monitoring of radioactive iodine tolerance, targeted therapy resistance and undifferentiated transformation processes.

Microfluidic chips boast a number of advantages, including their simplicity, miniaturization, and high throughput (51, 52). For instance, the single-cell immunoblotting (ieSCI) microfluidic chip integrates label-free inertial sorting with membrane enrichment technology to achieve efficient capture of CTCs at the single-cell level (e.g. changes in EpCAM expression) and dynamic analysis of their epithelial-mesenchymal transition (EMT) phenotypes changes (53). Recent studies have indicated the optical fluid cytometer (OFCM) is a sophisticated piece of equipment that integrates multi-level microfluidic technology with four-color fluorescence detection (54). This technology facilitates fully automated, high-throughput capture of CTCs, exhibiting exceptional recovery rates (>95%). The device also facilitates three-dimensional focusing of single cells and analysis of EMT phenotypes, including simultaneous detection of epithelial and mesenchymal markers, while processing 1.2 milliliters of whole blood per hour, providing a high-throughput solution for clinical applications (54). Surface-enhanced raman spectroscopy (SERS) integrated chips can not only capture CTCs through size screening but detect multiple single-cell membrane proteins using spectroscopically, orthogonal aptamer nanoprobes (55). A novel microfluidic platform addresses the limitations of traditional EpCAM-dependent methods by combining size-based sorting with dual-targeted magnetic beads (EpCAM/CD146) (56). Specifically, this approach improves the capture efficiency of heterogeneous CTCs in TC (including EpCAM-negative subpopulations) by 20% (56). Furthermore, it enables the non-invasive release of highly viable cells via matrix metalloproteinase-9 (MMP-9) hydrolysis (56). This integrated platform thus provides a more comprehensive LB solution for early metastasis warning, dynamic monitoring of drug-resistant clones, and high-throughput single-cell multi-omics research. Furthermore, The Ion Torrent PGM™ system, when employed in conjunction with the Ion AmpliSeq™ Cancer Hotspot Detection Panel (57), has been directly applied for the detection of gene mutations in CTCs from TC. This method effectively eliminates the whole-genome amplification step, which carries systemic bias, significantly improving mutation detection accuracy. In summary, various emerging microfluidic technologies have significantly advanced CTC detection through their advantages of miniaturization, high throughput, and multi-parameter analysis, painting a promising landscape for LB applications including TC. However, further research is required to translate these findings into clinical applications specific to TC.

### Clinical significance

The counting and dynamic changes of CTCs function can serve as direct biomarkers, which are helpful for evaluating surgical efficacy and predicting the recurrence risk. In TC, the core value of CTCs is currently more evident in postoperative dynamic monitoring of treatment efficacy and prognostic assessment (where changes in CTC counts over time hold greater predictive significance than baseline levels) (58). An analysis of CTCs in peripheral blood of patients with PTC before and after surgery revealed a significant decrease in postoperative CTC counts (preoperat: 5.0 cells/7.5 mL vs. postoperat: 2.5–3.0 cells/7.5 mL), which was particularly pronounced in patients with lymphovascular invasion, lymph node metastasis, or *BRAF^V600E^* mutation (59). This finding suggests that reduced CTC levels may serve as a biomarker for surgical success and a sustained decrease in CTCs is an indication of a lower risk of recurrence, while persistently high levels correlate with aggressive tumor characteristics (e.g., *BRAF^V600E^* mutation positivity), aiding in the early clinical identification of high-risk patients.

The distribution of heterogeneous CTC subpopulations provides a more comprehensive analysis of a tumor’s metastatic potential. CTCs comprise three distinct subtypes: epithelial CTCs (E-CTCs), mesenchymal CTCs (M-CTCs) and epithelial-mesenchymal CTCs (EM-CTCs) (60). The distribution patterns of these subpopulations are associated with tumor invasiveness and metastatic potential. For instance, an elevated M-CTCs or EM-CTCs ratio indicates a more aggressive tumor biology (60). Additionally, the presence of heterogeneous tumor cell clusters (particularly atypical CTC clusters) in the bloodstream is an early warning sign and a poor prognostic indicator of metastatic TC (60). These clusters form due to enhanced adhesion, mixed phenotypes, and interactions between CTCs and WBCs, involving key molecules. Not only does targeting these molecules that drive cluster formation and metastasis (some of which are existing drug targets) have therapeutic potential, but their expression levels can also serve as LB biomarkers for predicting patient survival (60). This provides new strategies for intervening in metastatic TC.

Currently, the field of CTC research is undergoing a fundamental shift from simple counting and phenotypic identification toward functional analysis and mechanism exploration. Research reveals that NanoVelcro chip technology enables the simultaneous capture of CTC clusters and individual CTCs without causing damage (61). Changes in the appearance of CTC clusters in advanced neuroendocrine tumors are significantly correlated with the response to and prognosis of peptide receptor radionuclide therapy (61). This finding suggests that monitoring both CTC clusters and individual CTCs in TC patients could provide a new, minimally invasive LB strategy for evaluating the effectiveness of targeted or radiation therapy. Recent researches have revealed significant advancements in single-cell multi-omics technology, covering genomics, epigenetics, transcriptomics, proteomics and metabolomics, have enabled in-depth molecular profiling of CTCs, which are key drivers of metastasis (62). This technology provides a novel approach to the study of the TC transfer mechanism. The integrated, multidimensional molecular characteristics of CTCs will also form the basis of a valuable LB biomarker system, optimizing the early diagnosis and targeted treatment of TC, as well as its prognosis.

In summary, the most promising prognostic biomarkers in functional CTC analysis converge on two aspects. Firstly, the M-CTC/EM-CTC ratio reflect the EMT state, providing a direct assessment of tumor invasiveness. Secondly, the detection of CTC clusters demonstrates the tumor’s capacity for clustered dissemination. From a technology transfer perspective, CTC subtyping techniques have reached a relatively mature stage, although there is still a need for broad consensus on clinical interpretation and standardization of results. Regarding biologically significant CTC clusters, research into efficient capture technologies, formation mechanisms and precise clinical prognostic value is still in its infancy, with genuine clinical applications only just beginning to emerge.

### Challenges of LB based on CTCs

The detection of CTCs poses multiple challenges, among which the most significant one lies in the difficulty of capturing and identifying low-abundance samples. Current technical limitations also render CTCs detection inadequate for identifying patients with micrometastases or early-stage cancer (63). Although CTCs can be used as a non-invasive biomarker for the dynamic monitoring of postoperative efficacy in PTC (58), its preoperative diagnostic value is limited (e.g. in distinguishing between follicular adenoma and follicular carcinoma), and it cannot replace ultrasound-guided FNAB as the gold standard for histopathological diagnosis. Currently, the lack of a unified CTC detection platform, standardized operating procedures, and result interpretation systems globally makes it difficult to compare and integrate data across different studies. At the same time, the complex CTCs detection process, which relies on specialized equipment and highly skilled operators, results in high costs. This significantly limits its adoption in routine clinical practice, particularly in regions with limited medical resources.

### Clinical translation perspective

The most promising applications of CTCs in TC include real-time monitoring of surgical efficacy and early risk stratification for recurrence. Prospective studies have indicated that CTC counts have the potential to assess therapeutic response following thyroidectomy, thereby providing a dynamic reflection of tumor burden (59). In addition, CTCs have the potential to support personalized treatment strategies, including the identification of resistance mechanisms and the assessment of metastatic potential, thereby providing a non-invasive foundation for clinical decision-making.

The presence of CTCs has been shown to have clear clinical relevance in solid tumors, such as breast and prostate cancer (64). However, in current LB research for TC, CTCs remain in an early stage of clinical translation as a key biomarker. The clinical validation of these biomarkers in TC is inadequate, with majority of the evidence being derived from limited prospective studies. For instance, in patients with PTC, CTCs counts as a non-invasive marker for assessing surgical efficacy, yet they have not gained widespread regulatory approval or achieved clinical standardization (59). There is a paucity of research supporting the validation of CTCs, with the majority of studies being of a small scale and not conducted across multiple centers (65).

The detection of CTCs presents a number of challenges, including low cell separation efficiency, complex operational procedures, and relatively high costs, particularly in distinguishing the circulating cancer stem cell subpopulation, which poses significant technical difficulties (50). Concurrently, alternative biomarkers such as ctDNA prove more readily implementable, as they do not require specialized cell separation methods, thus highlighting the relative disadvantage of CTCs in terms of cost-effectiveness. Moreover, the absence of unified technical standards and consensus in clinical applications further constrain their scalability and economic viability.

## Circulating tumor DNA

CtDNA originates from tumor cell apoptosis, necrosis or active release, ranging from 50 to 250 bp in size (66, 67). Its release level is influenced by tumor proliferation activity, volume and grade, and it can reflect the molecular characteristics of both the primary and metastatic sites simultaneously (68, 69). As a characteristic tumor marker, ctDNA can comprehensively present genetic variation information from tumor cell populations (70, 71). Changes in ctDNA can be used to predict the response to immunotherapy (72). Studies have shown that changes in ctDNA levels from before to during immunotherapy are significantly correlated with the patient’s response to treatment, and these changes occur earlier than the results of routine clinical imaging assessments (73). Changes in plasma ctDNA concentration are significantly correlated with tumor burden, tumor stage and therapeutic response (74). Current CtDNA testing methods have been extended to various body fluids (75). CtDNA testing primarily focuses on base substitutions, insertions and deletions (76). It should be noted that ctDNA has a short half-life of 16–150 minutes (77) and is highly fragmented. Therefore, the samples must be processed quickly to prevent degradation.

In early-stage PTC, the diagnostic efficacy of ctDNA remains subject to conflicting findings. Whilst studies indicate that plasma ctDNA biomarkers based on specific gene methylation can effectively distinguish PTC from benign thyroid nodules, suggesting diagnostic potential (78), other research has failed to conclusively establish an association between ctDNA detection and prognosis in patients with primary PTC (79). These conflicting results may stem from multiple factors. On the one hand, early-stage PTC typically exhibits low tumor burden, which can lead to reduced ctDNA release and detection difficulties. On the other hand, PTC possesses molecular heterogeneity and variations in aggressiveness, which can further influence ctDNA release levels and detection consistency (79). Additionally, discrepancies in sample types, pre-processing methods and detection sensitivity between studies may contribute to inconsistent outcomes.

### Separation and detection technology

Currently, ctDNA isolation and detection technologies based on different principles can be primarily categorized into four major types: digital polymerase chain reaction (dPCR), targeted NGS (next-generation sequencing), methylation analysis, and microfluidic and novel sensing technologies, as detailed in **Table 2**.

**Table 2:** Separation and detection techniques of ctDNA used in LB.

Technical classification	Specific technology	Application in TC	Clinical status	Core principle	Key metrics	Ref.
PCR-based molecular detection	ddPCR	Yes	Applied	Divide the sample into droplets for endpoint PCR, and perform absolute quantification through fluorescence signal statistics	Sensitivity: exceptionally high. Limitations: limited throughput.	(81–85)
	BEAMing technology	Yes	Applied	Liquid PCR connects magnetic beads, amplifying multiple templates simultaneously.	Sensitivity: high. Limitations: the technical process is relatively complex.	(81, 86, 87)
Targeted enrichment Technology based on NGS	Guardant360^®^	Yes	Retrospective studies	Enrich specific genomic regions in plasma ctDNA using hybridization capture technology, followed by high-throughput sequencing.	Limitations: targeted detection panel.	(89)
	CAPP-Seq	No	Under investigation	High-throughput sequencing is performed after enriching target genomic regions using selective probes.	Key advantages: suitable for tumor burden assessment and monitoring of MRD.	(91)
ctDNA methylation analysis	MCTA-seq	No	Under investigation	Construct sequencing libraries for amplified methylated CpG islands via multiplex PCR.	Sensitivity: high, particularly suitable for low-input ctDNA samples.	(110, 152)
Microfluidics and other emerging detection technologies	Microfluidic PCR Chip	No	Under investigation	Features 40,192 micro-holes and a bidirectional sample inlet design.	Sensitivity: capable of detecting abundance changes as low as 0.01%.	(120)

Polymerase chain reaction (PCR)-based detection methods, including droplet-based digital PCR (ddPCR), quantitative PCR and BEAMing technology, can detect low-frequency mutations with a mutation allele frequency (MAF) as low as 0.01% (80). In ddPCR, target sequences are absolutely quantified by dividing the sample DNA (which contains both the target and background DNA) into microdroplets (81, 82). These microdroplets are then amplified through endpoint PCR and statistically analysed based on the ratio of positive to negative microdroplets, as determined by the fluorescence signals (81, 82). ddPCR has several advantages, including simple operation, fast library construction, low sample requirements and ultra-high sensitivity (83, 84). For example, ddPCR exhibits high sensitivity and specificity in detecting cell-free DNA (cfDNA), accurately reflecting the tumor mutation status of ATC (85). However, its targeted detection characteristics limit its ability to analyze multiple genes in parallel (83, 84). BEAMing technology is an enhanced form of emulsion PCR (81, 86). It allows for the simultaneous amplification of multiple templates within a single tube reaction, as well as the effective recovery of target products via magnetic beads that are bound to the primers. For example, BEAMing technology can be used for minimal residual disease (MRD) detection in ATC (87). In order to surmount the limitations of conventional ddPCR throughput, a number of technological innovations have been developed. Nie et al. (88) used stepwise emulsification technology to create an array of nano-droplets on a glass chip for developing a multiplex dPCR platform, that can detect eight samples simultaneously, with a detection limit of 10 copies/μL and a dynamic range spanning four orders of magnitude.

The technology of targeted enrichment, based on NGS, can be defined as ultra-sensitive targeted detection achieved on a high-throughput platform. Plasma-based comprehensive NGS represents a promising NGS approach for TC. For instance, large-scale plasma ctDNA sequencing using the Guardant360^®^ platform has demonstrated that this method effectively maps the driver mutation profiles of various TC subtypes, including ATC, PTC, FTC, MTC, and others (89). Research indicates that, tagged-amplicon deep sequencing (TAm-seq) technology enables the detection of ultra-low-frequency mutations in plasma ctDNA with a sensitivity of > 97% and a lower limit of detection for mutant allele frequency of ~2% (90). Furthermore, cancer personalized profiling by deep sequencing (CAPP-Seq) technology provides a quantitative analysis of ctDNA. It has several advantages over traditional methods for assessing tumor burden, detecting MRD and monitoring early treatment responses (91). In recent years, NGS technology has been applied to endocrine tumor researches, with multiple findings on MTC published, which primarily used targeted sequencing strategies, with some also using Whole-Exome Sequencing (WES) methods (92–98). Although WES and Whole-genome sequencing (WGS) can achieve whole genome coverage, their sensitivity for detecting low-frequency variants is significantly limited compared with targeted sequencing (80). In summary, the aforementioned high-sensitivity detection technology, which enables dynamic quantitative monitoring of ctDNA, has emerged as a potentially significant tool for the detection of MRD and prediction of recurrence following TC surgery.

Methylation profiling of ctDNA has become an effective tool for the early diagnosis, monitoring of recurrence and assessment of prognosis of various cancers (99–101). As the promoter region of tumor suppressor genes is almost completely unmethylated in healthy individuals, the detection of abnormal methylation in CTCs and ctDNA is highly specific (102–104). A multicenter study revealed that DNA methylation undergoes progressive demethylation during the metastasis of DTC, with 60% methylation convergence observed in metastatic lesions of different subtypes (PTC/FTC) (105). Based on the 156-CpG methylation profile of the primary tumor, DM risk can be accurately predicted (105). In some studies, it has been shown to be significantly more sensitive than ctDNA mutation analysis in detecting MRD (106). As the most reliable method for DNA methylation profiling, bisulfite sequencing enables precise quantification at single-nucleotide resolution (107). Several well-established DNA methylation profiling technologies have been developed, including whole-genome bisulfite sequencing (WGBS) (108), reduced representation bisulfite sequencing (RRBS) (109), targeted enrichment approaches, methylation CpG tandem amplification and sequencing (MCTA-seq) (110), and methylation arrays. Non-bisulfite conversion approaches for DNA methylation analysis include antibody enrichment and restriction endonuclease techniques. Among antibody-based methods, methylated DNA immunoprecipitation (MeDIP) utilizes antibodies specific to 5-methylcytosine (5mC) to selectively enrich methylated DNA fragments, with methylation levels subsequently determined by quantifying differential fluorescence intensity (111). Notably, Cell-free MeDIP and high-throughput sequencing (cfMeDIP-seq), an adaptation for cfDNA, demonstrates high sensitivity in detecting ctDNA methylation patterns, enabling effective identification and classification of diverse cancer types (112).

Microfluidic systems that integrate PCR and RNA sequencing technologies are widely used for detecting gene mutations (113–117). Of these, the recently reported high-throughput, single-cell RNA sequencing microfluidic system allows for the analysis of large numbers of samples in parallel (118). Additionally, microfluidic platforms with multiple detection functions can analyze multiple genetic targets simultaneously, which significantly improves detection efficiency (118). Ou et al. (119) developed the integrated comprehensive droplet digital detection, an integrated droplet digital detection system, to further enhance detection throughput. This system combines microfluidic droplet partitioning and multiplex PCR reactions with rapid, three-dimensional, large-volume droplet scanning and counting technology. In mutation allele detection, the system demonstrated 100% specificity, with sensitivity ranging from 0.00125% to 0.005% and a false positive rate of 0% (119). To enable highly sensitive detection of ctDNA, researchers have developed a microfluidic PCR chip containing 40,192 micropores and featuring a bidirectional sample inlet area (120). This chip can detect changes as small as 0.01%. By contrast, Yu et al. (121) designed a chip based on the self-partitioning sliding principle. Its “chain-of-pearls” continuous microfluidic channels enable the spontaneous generation of droplets. This simple platform does not require complex channel design or external pump valve systems and is easy to operate (121). The detection of ctDNA poses unique challenges due to its short half-life and extremely low abundance in early-stage cancers. Researchers have developed an ultrasensitive electrochemical biosensor that can rapidly detect ctDNA in whole blood (122). The DNA modified gold-coated magnetic nanoparticles (DNA-Au@MNPs) sensor can specifically recognize short-chain and long-chain DNA targets (122). Moreover, it can detect ctDNA in whole blood within 20 minutes, offering a new, highly sensitive, minimally invasive strategy for early TC diagnosis.

### Clinical significance

CtDNA analysis provides a non-invasive basis for molecular subtyping of TC. In cases of TC, the v-Raf murine sarcoma viral oncogene homolog B (BRAF) gene is the most frequently mutated, with an approximate mutation rate of 76.0%. This is followed by rearranged during transfection (RET) rearrangements (7.6%) and rat sarcoma (RAS) driver mutations (4.1%) (123). The *BRAF^V600E^* mutation is a highly valuable biomarker for LB, due to its high specificity in TC and its role as a target for molecularly targeted therapies (124). *BRAF^V600E^* was detected in 27.2% and 35.7% of ATC and PTC cases, respectively (89). However, it was not detected in any FTC cases (89). In samples where RET fusions were detected in plasma and the tumor tissue clearly reported fusion partners, the consistency rate of fusion partners identified based on LB and tissue biopsies ranged from 81% to 89%, with coiled coil domain containing 6 gene and nuclear receptor coactivator 4 being the most common RET fusion partners (125, 126). The respective detection rates of RAS mutations in ATC, PTC and FTC were 18.5%, 11.9% and 62.5% (89). An analysis of 17 studies involving 2,552 TC patients revealed that the overall incidence of RAS mutations was 35.4% (95% confidence interval (95% CI): 22.7–50.7) (127). Among these, NRAS mutations were the most prevalent (69.47%, 95% CI: 66.15%–72.66%), followed by HRAS (25.83%, 95% CI: 22.77%–29.14%) and KRAS (6.92%, 95% CI: 5.27%–9.04%) mutations (127).

In terms of prognostic risk stratification, comprehensive mutational profiles revealed by dynamic monitoring of ctDNA have emerged as the most promising molecular tool for assessing tumor metastatic potential. The genetic profile of ctDNA, particularly combinations of specific mutations, serves as a powerful prognostic prediction tool. BRAF mutations are associated with a poor prognosis in PTC and are commonly found in ATC with an aggressive, undifferentiated phenotype (128–130). PTC accompanied by a *BRAF^V600E^* mutation is usually associated with a significant reduction in thyroid differentiation marker expression (131). This makes the cancer more resistant to radioactive iodine treatment and associated with a poorer prognosis (131). Analysis of genetic background revealed that the rs2853669 TT genotype was significantly associated with an increased risk of PTC recurrence (adjusted hazard ratio [HR] = 2.12, 95% CI: 1.10–4.12) (131). However, this association was not statistically significant in the TC/CC genotype context (adjusted HR = 1.17, 95% CI: 0.56–2.41) (131). Of particular importance are patients who carry all three of the following mutations: *BRAF^V600E^*, TERT promoter and rs2853669 TT. These patients have a recurrence rate of up to 76.5%. In contrast, patients with neither of these mutations and the TC/CC genotype had a recurrence rate of only 8.4%. There is a significant difference in recurrence risk between these two groups (HR = 13.48, 95% CI: 6.44–28.21) (131). A meta-analysis of 11 PTC studies demonstrated the diagnostic performance of ctDNA-based *BRAF^V600E^* mutation detection as follows: combined sensitivity was 56% (95% CI: 36%-74%), specificity was 91% (95% CI: 84%-95%), and diagnostic odds ratio was 12 (95% CI: 4.09-33.11) (133). The *BRAF^V600E^* mutation shows promise as a tool for monitoring disease progression in advanced TC (124). It is worth noting that the presence of RAS mutations is significantly associated with an increased risk of DM and death in patients with TC, suggesting that they may serve as prognostic markers (134). TP53 mutations are uncommon in DTC, but are significantly more prevalent in ATC (134). Research data from Japan shows that the detection rate of TP53 mutations in ATC patients is significantly higher than in patients with advanced non-ATC TC (the respective rates being 82.3% and 11.8%, *P* < 0.001) (135).

In terms of treatment guidance and monitoring, ctDNA’s core value lies in its ability to provide a basis for targeted therapy and enable the dynamic assessment of treatment efficacy. Longitudinal quantitative monitoring of ctDNA levels is the most promising method for monitoring dynamic treatment responses, due to its short half-life and ability to reflect changes in tumor burden in near real time (136). The core clinical significance of ctDNA lies in its ability to provide a basis for targeted therapy. Research indicates that, *BRAF^V600E^* driver mutations are frequently found in DTC and ATC, and BRAF inhibitors targeting this mutation have demonstrated clinical efficacy (135). In addition, the detection rate of RET gene alterations in patients with PTC is 10–20%, whereas in patients with MTC, the detection rate is almost 100% (137). In response to the challenges of detecting rare NTRK gene fusions in TC, experts recommend using NGS-based DNA/RNA sequencing for initial screening of patients with advanced, unresectable, high-risk or radioactive iodine-refractory disease (138). Positive results can directly guide the use of myosin light chain kinase inhibitors (such as larotrectinib and enzurtinib) (139). This underscores the critical value of LB techniques like ctDNA fusion detection in non-invasively identifying patients eligible for targeted therapies (139).

### Challenges of LB based on ctDNA

The challenges associated with analyzing early-stage cancer ctDNA stem from its short half-life and extremely low abundance (122). The low detection rate of ctDNA in TC may be related to tumor proliferation activity, volume, and histological subtype (68). Research confirms that tumor proliferation indices correlate positively with ctDNA detection sensitivity, while DTC typically exhibit low proliferative states, directly explaining this phenomenon (68). Compared to other solid tumors (e.g., gastric cancer, lung cancer), TC demonstrates a significantly lower ctDNA detection rate (140). For instance, a study in gastric cancer reported a ctDNA detection rate of 7.7%, surpassing the 2.6%-4.4% rate achieved by tissue biopsy (140). In contrast, comparable detection levels have not yet been achieved in TC (140).

The sensitivity and breadth of detection are constrained by technical limitations. Tissue testing remains the gold standard for the majority of molecular typing of cancers, as ctDNA testing has limited ability to identify fusion events and copy number variations (141). However, in circumstances where expeditious results are imperative for clinical purposes or tissue biopsy is not a viable option, ctDNA testing can be utilized as a conventional alternative (141). Despite the capacity of ctDNA to detect genomic mutations in TC (e.g. *BRAF^V600E^*), conventional methods (e.g. ddPCR) are susceptible to false negatives in low-abundance samples (142). Integrated nucleic acid separation and analysis technologies based on microfluidic systems, compared to traditional methods, currently offer superior cost-effectiveness, pollution control and processing speed. However, this technology usually extracts multiple nucleic acids non-specifically, which may affect the representativeness of the sample (143). Therefore, establishing standardized sample collection and processing procedures is necessary to build a reliable and efficient microfluidic platform. Although current research has focused primarily on developing detection functional units, microfluidic chips that integrate the extraction and detection of ctDNA, especially those based on dPCR, remain scarce (45).

Although ctDNA methylation profiles hold promise as LB biomarkers for applications spanning early detection, therapy monitoring, and prognosis prediction in TC (144), there is still a lack of solid evidence for clinical validation of ctDNA. A small-sample study of 22 patients with localized PTC suggests that preoperative detection of ctDNA is limited and its correlation with postoperative prognosis remains to be established (79). Its clinical value in TC remains unclear due to biological heterogeneity and technical limitations, particularly in the case of primary PTC, for which there is still insufficient evidence to support its use (79, 145). Even in PTC patients without distant metastasis, the prognostic predictive value of ctDNA remains unclear, suggesting that its release may be regulated by the tumor micro-environments or metabolic reprogramming (79, 146). In addition, other limitations include: difficulties in ensuring technical reproducibility and controlling batch effects, a paucity of multi-center validation data, complex operational procedures and high costs per sample, the inherent low signal-to-noise ratio stemming from minimal ctDNA abundance in early-stage cancers, and the current lack of standardized testing frameworks (144, 147). These challenges are generally addressed by existing detection methods that require extremely high sequencing depth, which significantly increases detection costs (148). Emerging technologies, such as pyrimidine-dependent UV-based minor-allele enrichment (PD-UVME), have enhanced the sensitivity of detection. However, concerns pertaining to standardization of procedures and cost control persist (108).

### Clinical translation perspective

The clinical validation of ctDNA in TC as a biomarker remains in its infancy, with primary evidence derived from retrospective cohort studies and investigations targeting specific subtypes (e.g. ATC and PTC) (79, 87). The most promising recent applications of ctDNA include the early detection of disease progression or recurrence in aggressive, high-mortality ATC, thus providing the potential for timely adjustment of treatment strategies (87). In cases of advanced or refractory TC, where tissue samples are either inaccessible or insufficient, ctDNA serves as a complementary method to identify targetable genetic alterations, thus informing precision therapy (87). Following surgical resection, particularly in high-risk patients, this method is utilized for the assessment of MRD status, with the objective of identifying patients who are at high risk of recurrence (87). This information is then used to guide decisions regarding adjuvant treatment or to intensify monitoring (89). By longitudinally monitoring the dynamic changes in ctDNA levels, it is possible to indicate treatment response or resistance earlier than conventional imaging. This, in turn, facilitates real-time adjustments to therapeutic regimens (149, 150).

However, in comparison to tissue biopsy, the sensitivity and specificity of ctDNA testing in TC require extensive validation through larger-scale prospective studies (89). The technical intricacy and considerable expense associated with ctDNA testing (particularly NGS-based methods) serve to limit its extensive implementation in routine clinical practice and screening programs (89). Robust cost-effectiveness analysis data are currently lacking. Furthermore, ctDNA release levels in TC - particularly in early-stage or low-burden disease – may be low, placing exceptionally high demands on the sensitivity of detection technologies (122). The spatiotemporal heterogeneity of tumors may also compromise the representativeness of ctDNA detection. At present, there is an absence of standardized protocols for the collection, storage, processing, and analysis of ctDNA samples (89, 151). This poses challenges to the comparability of results across different platforms and detection schemes. The development of clearer clinical guidelines is imperative for the interpretation of low-level ctDNA or negative results, particularly in cases of low tumor burden.

## Exosomes

Exosomes are nano-sized (30–150 nm in diameter) double-membrane vesicles (153). They are universally secreted by all cell types, released into extracellular space and circulation through the exocytosis of multivesicular bodies, and ubiquitously present in all bodily fluids (154). Its accessibility make exosomes highly attractive biomarkers for LB, facilitating longitudinal monitoring of disease progression. Research indicates that tumor cells exhibit substantially elevated exosome secretion compared to normal cells, even up to 10-fold (154). The diagnostic potential of exosomes in LB is superior to that of ctDNA and CTCs, specifically reflected in their abundance far exceeding CTCs, and the sources of biomarkers such as RNA, DNA, proteins, lipids and metabolites they carry are more diverse than ctDNA and the information is more comprehensive (154, 155). These molecules transmit biological information from tumor cells, and also participate in the regulation of tumor micro-environments (156). Molecular markers, such as vesicle surface proteins, can indicate the tissue of origin and the tumor information they encapsulate is more stable in bodily fluids, making it less prone to degradation. Exosomes exert their functional effects primarily through exosome-mediated intercellular communication and interactions with the micro-environments (156). Applying multi-omics approaches (such as genomics, proteomics, lipidomics and metabolomics) to analyze exosomes content can provide deeper insights into disease states (157). In particular, analyzing RNA of exosomes can help to identify genes that are expressed differently and deepen our understanding of the mechanisms of disease progression (158). Multiple studies have confirmed the potential value of exosomes in diagnosing and managing thyroid diseases (including malignant tumors), as well as their ability to serve as novel tumor biomarkers (158–163).

### Separation and detection technology

The research technology system for exosomes has been continuously evolving around two major components: isolation and purification, as well as downstream detection and analysis, as shown in **Table 3**.

**Table 3:** Isolation and detection techniques for exosomes in LB.

Main sections	Technology category	Specific technology	Application in TC	Clinical status	Core principle	Key metrics	Ref.
Separation and purification technology	Based on physical properties	UC	No	Gold standard	Exosomes are precipitated and separated from biological fluids under high-speed centrifugal force based on particle size, density, and sedimentation coefficient.	Limitations: time-consuming; potential damage to exosomes due to strong shear forces.	(166)
		TFF	No	Under investigation	The sample flows tangentially across the filter membrane surface, reducing membrane clogging and enabling efficient filtration and concentration.	Recovery rate/throughput: significantly enhances separation efficiency and yield (92.5 times higher than UC), scalable.	(168, 211)
	Based on affinity properties	IAC	No	Under investigation	Capture by binding antibodies to surface-specific antigens on exosomes.	Specificity: high, yielding high-purity exosomes. Limitations: high cost, low recovery rate, demanding operational requirements.	(170–172)
		ExoRNeasy serum/plasma kit	Yes	Applied	Purify total exosomal RNA from serum/plasma samples based on membrane affinity binding principles.	Application: widely employed for exosomal RNA extraction, compatible with downstream analyses. Limitations: primarily targets nucleic acids rather than intact exosomes.	(173, 174)
		V-functionalized microfluidic chip	No	Under investigation	Utilizing a 3D serpentine topology, V-functionalized targets phosphatidylserine on the surface of exosomes to enhance capture.	Capture efficiency: high capture efficiency for exosomes derived from cancer cells (approximately 90%).	(180)
Detection and biomolecular analysis technologies		qRT-PCR	Yes	Applied	Following exosome RNA extraction, specific miRNAs were quantified via reverse transcription and quantitative real-time PCR.	Sensitivity: high, serving as a widely adopted benchmark method.	(182, 183)
		ECL-RET biosensor	Yes	Applied	Through synergistic combination of target recovery amplification and efficient Zr-PTC electrochemical luminescence labeling.	Sensitivity: ultra-high (detection limit 0.69 fM), for the detection of the thrombocytopenia biomarker miR-146b-5p.	(186)
		miRNA (CirsEV-miR) classifier	Yes	Under investigation	Diagnostic model based on specific miRNA profiles.	Diagnostic performance: demonstrated robustness (AUC > 0.84), correlated with tumor burden, and exhibited high specificity for FTC	(187)
		Label-Free SERS-LB	No	Under investigation	Mix FNAB wash solution with silver nanoparticle colloid in a quartz capillary to directly obtain SERS spectra, which are then analyzed using a CNN.	Diagnostic accuracy: 88.1% accuracy, 87.8% sensitivity. Advantages: avoids bias and subjective interpretation.	(192)

The isolation of TC cells is primarily achieved through the utilization of differential centrifugation and precipitation methods (164, 165). Ultracentrifugation (UC) is often considered the gold standard for separating exosomes due to its reliability and versatility (166). As a derivative technology of UC, differential-gradient (DG) centrifugation is considered a standard method for the separation of extracellular vesicles (EVs) (167). Furthermore, ultrafiltration typically involves the use of ultrafiltration membranes with a molecular weight cut-off (MWCO) of 10–100 kilodaltons (kDa), often as an initial step to concentrate EVs from large volumes of raw samples for subsequent purification (168). Another study indicates that tangential flow filtration (TFF) significantly improves the efficiency and yield of exosome separation. For example, applying TFF to separate exosomes derived from human umbilical cord mesenchymal stem cells (UCMSCs) increased the yield by a factor of 92.5 compared to the UC method (168). TFF is a scalable and efficient technology that is particularly well-suited to the isolation and enrichment of exosomes from large volumes of cell culture medium (168). There are significant differences in the efficiency and quality of products among commercially available exosome separation kits. Some studies have shown that, compared to polymer-based kits such as ExoQuick™ kit achieves a higher yield and produces higher-quality, purer exosomes (169).

Due to their high selectivity, immunoaffinity capture (IAC) technologies are often used to prepare high-purity exosomes (170). The IAC employs antibodies to capture exosomes by binding to surface-specific antigens (171). Nevertheless, the technology’s high cost, low recovery rate, and demanding operational requirements limit its application in clinical sample analysis (172). The exoRNeasy Serum/Plasma Kit, which is based on membrane affinity binding principles, has a wide range of applications for purifying total exosomal RNA and has been directly employed in exosome isolation studies for TC (particularly PTC) (173, 174).

Microfluidic systems are ideal for separating exosomes from other nanoscale particles thanks to their low cost, high speed and precision (175). Liu et al. (176) proposed the exosome total isolation chip (ExoTIC), a label-free separation method based on size differences. Due to its simple, modular structure, the chip can easily process a variety of samples, such as cell culture media, saliva, serum, urine and plasma (177). The ExoTIC device offers advantages over polyethylene glycol (PEG) precipitation methods (including the ExoQuick™ kit) and UC when it comes to extracting exosomes from serum and other bodily fluids (178). Notably, this device can efficiently isolate exosomes from small samples, such as 10–100 μL of fingerstick blood (179). Some research data show that the yield of exosomes obtained using ExoTIC is significantly higher than that obtained using UC (up to several or even hundreds of times higher), making ExoTIC an ideal choice for point-of-care testing based on exosomes (179). Kang et al. (180) developed an Annexin V-functionalized microfluidic chip with 3D serpentine topology to enhance exosome capture via phosphatidylserine targeting. The device achieved 90% separation efficiency for cancer-derived exosomes (vs. 38% for normal sources) in spiked samples, demonstrating potential for TC exosome isolation (180). Conjugated 1-pyrenecarboxylic acid (COL-Py) based self-assembled magnetic nanoclusters (CMNCs) enable high-purity exosomes isolation validated by Western blotting, preserving structural integrity for accurate low-abundance biomarker detection (181). Their reusability reduces costs and enhances scalability, providing a robust platform for large-scale TC screening and dynamic monitoring via LB.

The qualitative and quantitative analysis of isolated exosomes and the biomolecular information they carry is crucial for realizing their clinical value. MiRNA is the nucleic acid biomarker that has been studied most extensively in exosomes, and analytical techniques are evolving towards ultra-sensitivity and high-throughput capabilities. Presently, quantitative reverse transcription PCR (qRT-PCR) has been directly applied for the detection of microRNAs in TC exosome samples (182, 183). Liu et al. (184) developed an enzyme-free electrochemical sensor that uses programmable tetrahedral supercharged tetrahedral DNA nanolabel-based electrochemical sensor (eTDN) probes to enable direct, blood-based exosomal miRNAs (Exo-miRs) profiling analysis. Solution-phase target recognition, amplified signal transduction via charge-enhanced reporter adsorption, and electroneutral peptide nucleic acid enabled background suppression collectively deliver 34 aM detection limits with single-base specificity, successfully discriminating breast cancer patients. It may also be applicable for early detection or MRD monitoring in malignant tumors such as TC. *In situ* detection of Exo-miRs, an attractive non-invasive biomarkers, bypasses tedious extraction steps, enhancing diagnostic accuracy (185). To address low target abundance and poor probe permeability, a multi-branched localized catalytic hairpin assembly (MLCHA)-photonic crystal (PC) biochip was engineered: rigid MLCHA probes penetrate exosomal membranes to trigger *in situ* cascade amplification, while PC bandgap resonance further amplifies fluorescence, achieving ultrahigh sensitivity (185). This platform quantifies exo-miRs and discriminates cancer states, offering transformative potential for non-invasive TC diagnosis and monitoring. An electrochemiluminescence (ECL)-resonance energy transfer biosensor leveraging toehold-mediated strand displacement reaction (TSDR) amplification and zirconium-porphyrin-based tetracarboxylate (Zr-PTC) metal-organic framework signaling enables ultrasensitive detection of TC biomarker miR-146b-5p (186). This design synergistically combines target recycling amplification with efficient Zr-PTC ECL tags, achieving a 0.69 fM detection limit alongside excellent reproducibility and stability. The circulating small EVs miRNA (CirsEV-miR) classifier demonstrated robust diagnostic performance in training (n = 41, AUC = 0.924) and multicentric validation cohorts (n = 150, AUC = 0.844), correlating with tumor burden and showing FTC-specific elevation in models and tissue small EVs (187). Its treatment-resistant stability enables longitudinal monitoring, while clinical integration optimizes surgical decisions for personalized FTC management.

The exosome detection method via the ultrafast-isolation system (EXODUS), leveraging negative-pressure oscillation and dual-coupled resonator-driven membrane vibration, enables ultrafast, label-free exosome purification with superior yield/purity (188). Successfully applied to > 100 clinical urine samples, its output facilitates high-resolution RNA sequencing. This automated platform provides robust technical support for large-scale screening of exosomal RNA biomarkers in LB (e.g., urine/blood), potentially enabling its application TC diagnostics. It is evident from further studies that the ExoChip microfluidic platform facilitates straightforward and effective analysis of serum exosome samples (189). By preserving exosomal RNA integrity, it enables downstream miRNA profiling (e.g., OpenArray), critical for discovering TC-specific biomarkers (189). Its proven capability to detect exosome abundance differences and analytical compatibility positions it as a potent tool for LB research of TC. Additionally, the method based on graph convolutional neural network (CNN) and conditional random field (GCNCRF) for predicting human lncRNA–miRNA interactions, named GCNCRF, integrating graph convolutional networks with conditional random fields, achieves superior prediction of human lncRNA-miRNA interactions (5-fold CV AUC: 0.947), outperforming existing methods (190). This high-accuracy approach efficiently mines latent interactions from complex networks, accelerating the construction of functional noncoding RNAs (ncRNA) regulatory networks in TC.

An electrochemically activated nano-platform enables direct, rapid detection of exo-miRs in biofluids with high sensitivity (low detection: 0.5 nM) and specificity (191). Critically, weak electrical stimulation enhances exosome secretion and miRNA loading, achieving active enrichment (191). This SERS-based *in situ* detection method may drive breakthroughs in biopsy technology for non-invasive screening, molecular subtype classification, and dynamic monitoring of TC. In addition, to overcome the limitations of traditional FNAB cytology, such as labor-intensity and false negative rate, a label-free SERS-LB analysis technique was proposed to detect the mixture of FNAB lavage solution and Ag nanoparticle colloids in quartz capillaries (192). A CNN-based deep learning model trained on 36 clinical specimens achieved 88.1% accuracy, 87.8% sensitivity, and 0.953 AUC, eliminating staining/subjective interpretation (192). This cost-effective strategy provides a new approach for the rapid screening of TC and the diagnosis of uncertain nodules.

### Clinical significance

In the field of LB for TC, analyzing specific miRNA profiles based on exosomes (such as the combination of miR-16-2-3p and miR-223-5p) is considered one of the most promising approaches to achieve an early diagnosis and distinguish between benign and malignant thyroid nodules. This is primarily because these extracellular molecules possess unique molecular markers characteristics that make them ideal targets for non-invasive diagnosis and differential diagnosis of TC. With the continuous deepening of related research, a series of miRNA biomarkers with considerable diagnostic potential have been successfully identified one after another. For example, plasma exosomal miR-16-2-3p, miR-223-5p, miR-34c-5p, miR-182-5p, miR-223-3p, and miR-146b-5p are significantly downregulated in thyroid nodule patients vs. healthy controls, serving as potential screening biomarkers (193). Among these, miR-16-2-3p and miR-223-5p can discriminate malignant from benign nodules, while miR-16-2-3p, miR-223-5p, miR-101-3p, and miR-34c-5p achieves superior diagnostic accuracy for PTC. Furthermore, a three-plasma-miRNA signature (miR-346/miR-10a-5p/miR-34a-5p) verified in a multicenter Chinese cohorts can effectively distinguish PTC from healthy controls (AUC: 0.816–0.926) and benign diseases (AUC = 0.877), enabling high-accuracy LB method for the preliminary screening of TC (194). To address the diagnostic challenges of FTC, a novel strategy have proposed enriching thyroid peroxidase-positive (TPO^+^) exosomes from plasma and analyzing their Let-7 miRNA profiles, achieving the discrimination of FTC (AUC: 0.77–0.84). This method effectively reduces the interference of plasma heterogeneity by focusing on tissue-derived exosomes, providing a new idea for the precise diagnosis of FTC.

Furthermore, exosomes can be used to indicate the malignant progression of tumors, assess prognosis and monitor the response to treatment. Plasma levels of miR-25-3p and miR-451a are significantly elevated in PTC patients, mirroring tumor tissue expression and decreasing postoperatively (195). Hypoxic tumor micro-environments in PTC drive cancer cells to secrete exosomes enriched with miR-221-3p, which is elevated in patient plasma, tissues, and cells (196). Upon recipient cell uptake, miR-221-3p promotes tumor progression by targeting Zinc Finger AN1-Type Domain 5 (ZFAND5) to enhance proliferation, migration, invasion, and epithelial-mesenchymal transition (196). This pathway positions plasma exosomal miR-221-3p as a prognostic biomarker and a therapeutic target for PTC. Dai et al. (197) showed that the overexpression of miR-221 should be considered a risk factor for the recurrence of PTC (HR: 1.41; 95% CI: 1.14–1.95, *P* = 0.007). Postoperative decline of miR-21/221/146b/181b positions them as dynamic biomarkers for disease activity monitoring (198).

Tumor cells communicate with other cells in the micro-environments via exosomes, creating an ecosystem that promotes tumor growth and metastasis. For example, hypoxic PTC-derived exosomes carry enriched miR-181a, which upon human umbilical vein endothelial cells (HUVECs) uptake drives angiogenesis via stimulated endothelial proliferation and tube formation (199). This hypoxia-exosome-miRNA axis mediates tumor-endothelial crosstalk in TC (199). Exosomal Dedicator of cytokinesis 9 antisense RNA2 (DOCK9-AS2) lncRNA is specifically enriched in plasma exosomes of PTC patients, predominantly derived from PTC stem cells (PTC-CSCs) (200). Functionally, DOCK9-AS2 drives malignant progression by enhancing proliferation, migration, invasion, EMT, and stemness in recipient cells (200). Critically, PTC-CSCs deploy exosomal DOCK9-AS2 to disseminate stem-like traits across the tumor micro-environments, revealing a novel mechanism of CSC-driven niche remodeling (200). Research has found that the long non-coding RNA HOX transcript antisense RNA (HOTAIR) is significantly overexpressed in PTC tissue and plays a key role in carcinogenesis (201). Through an integrated analysis of TCGA database and subsequent experimental validation, researchers identified the homologous box gene Distal-less homeobox 1(DLX1) as a key downstream target of HOTAIR (201). Not only does the HOTAIR-DLX1 regulatory axis play a significant role in the development of PTC, but its key components (high HOTAIR expression and low DLX1 expression) can also serve as important tissue biomarkers for assessing disease progression and prognosis in PTC (201).

### Challenges of LB based on exosomes

The efficient and accurate separation of high-purity exosomes remains a major technical bottleneck due to their nanoscale size and significant heterogeneity (202). The composition of EVs in plasma is complex, including various subtypes such as exosomes and microvesicles, which differ in size, origin and contents. The inherent heterogeneity of EVs in plasma further hinders the development of new diagnostic methods and reduces the predictive value of these methods in distinguishing between benign follicular adenoma and malignant FTC (203). Currently, no separation method can simultaneously meet the requirements of high purity and recovery rates, simple operation and low cost. For example, although the UC-based isolation method has a relatively high purity, it is cumbersome to operate, which can easily lead to the aggregation of exosomes, reduced yield, and the introduction of a large amount of protein aggregates and lipoprotein contaminant, thereby restricting the development of high-quality exosome research (166, 204). There are significant variations in the separation efficiency and quality exosomes among various exosome isolation kits available on the market (204, 205). Studies have shown that the EVs obtained using different separation methods (e.g. UC, SEC and commercial kits such as ExoQuick/ExoEasy) exhibit significant differences in their biophysical properties, proteome and glycome composition (206). These differences directly affect the comparability of downstream analysis results making it difficult to directly integrate and compare data from different studies. For instance, UC and commercial kits perform differently in terms of separation purity, RNA recovery rate, and consistency of sequencing data obtained, further exacerbating the inconsistency in research results (207).

### Clinical translation perspective

Exosome-based tumor markers (e.g. specific microRNAs, proteins, etc.) are currently in the early stages of clinical validation. For instance, although studies have indicated an association between the presence of the microRNA-222-3p and BRAF mutations (207), and these biomarkers have potential applications, such biomarkers have yet been implemented in a wide range of routine clinical context (186, 208). The clinical translation of this technology is hindered by several obstacles, primarily the absence of standardized procedures for exosome isolation, characterization, and preparation. The intricacies inherent in high-sensitivity detection techniques, the necessity for highly purified exosome preparations, and the challenges associated with large-scale production all collectively impact the cost-effectiveness and accessibility. The primary application prospects in this field are concentrated in two areas. Firstly, the development of highly sensitive diagnostic or prognostic tools based on specific exosomal biomarkers (such as miR-222-3p), which could serve as an effective supplement to existing methods like ultrasound and fine-needle aspiration (186, 207); Secondly, engineered exosomes serve as targeted therapeutic carriers or tools for modulating the immune microenvironment, offering novel strategies for cancer treatment (209, 210).

## Discussion

The paradigm of TC management is undergoing a pivotal shift, propelled by the rapid advancement of LB. This review has delineated the significant progress in the three cornerstone biomarkers—CTCs, ctDNA, and exosome. As shown in **Table 4**, different biomarkers have different applications: CTCs provide a complete cellular functional model for investigating tumor metastasis and heterogeneity. CtDNA, owing to its high temporal resolution, plays a crucial role in detecting driver mutations (such as *BRAF^V600E^* and RAS), monitoring MRD and tracking clonal evolution. Exosomes carry abundant functional RNAs and proteins that reflect intercellular communication and regulatory networks. However, translating these biomarkers into reliable clinical tools requires a systematic evaluation of their technical characteristics and applicable scenarios.

**Table 4:** Clinical characterization of LB biomarkers in TC.

Biomarkers	Specific indicators	Sensitivity in early disease	Prognostic value	Clinical readiness level	Ref
CTCs	Dynamic counting	It can be detected in early PTC, but technical limitations result in overall low sensitivity.	High. A postoperative decrease correlates with surgical success; persistently high levels correlate with a high risk of recurrence and aggressive features (such as *BRAF ^V600E^* mutation).	Exploratory phase. Limited validation in clinical trials, insufficient standardisation, and questionable cost-effectiveness; core value lies in postoperative dynamic monitoring.	(58, 59)
	Subtype analysis (E-CTC, M-CTC, EM-CTC)	An elevated M-CTC/EM-CTC ratio indicates the presence of a more aggressive tumor cell subpopulation and may be utilised for early risk identification.	Elevated M-CTC or EM-CTC ratios serve as strong predictors of tumor invasiveness and high metastatic potential.	Research phase. Phenotype recognition technology is approaching maturity, but clinical validation and standardised interpretation remain in their early stages.	(60)
	Cell clusters	The emergence of CTC clusters serves as an early warning sign of metastasis.	Extremely high. The presence of CTC clusters constitutes an independent prognostic factor for poor outcomes and is significantly associated with the risk of metastasis.	Frontier exploration. Its formation mechanisms and potential as therapeutic targets are research hotspots, with clinical translation only just commencing.	(60, 61)
ctDNA	BRAF ^V600E^	Detection rates are high in advanced ATC/PTC (27.2%/35.7%); sensitivity is limited in early-stage/low-burden disease (pooled sensitivity of 56% in meta-analysis).	Extremely high. Strongly associated with an aggressive phenotype and poor prognosis; in combination with TERT promoter mutations, it elevates the risk of recurrence by 13.48-fold.	Preliminary application (advanced stage). This represents a key biomarker for prognostic stratification and targeted therapy guidance in advanced-stage TC, supported by substantial evidence.	(87, 89, 124, 132, 133)
	RET fusion	The highest detection rate was observed in FTC (62.5%), while PTC and ATC recorded rates of 11.9% and 18.5% respectively.	High. This is the core driver event in MTC, present in almost 100% of cases; in PTC, it is associated with specific pathological subtypes.	Therapeutic guidance application. A positive test result directly guides the use of RET inhibitors, demonstrating clear clinical translational value in advanced-stage patients.	(89, 125, 126, 137, 139)
	RAS mutation (NRAS, HRAS, KRAS)	The fusion of LB and organisational detection exhibits high companion consistency.	High. It is significantly associated with increased risks of distant metastasis and mortality, making it an important prognostic marker.	Validation phase. Its value is well-established in specific subtypes (such as FTC) and prognostic assessment, but data are still required for broad clinical guidance applications.	(89, 127, 134)
	Other (e.g. TP53, TERT)	The detection rate of TP53 in ATC was significantly higher than in non-ATC (82.3% vs 11.8%), demonstrating subtype specificity.	Extremely high. TERT promoter mutations constitute a potent adverse prognostic factor; TP53 mutations correlate with the exceptionally aggressive nature of acute medullary leukaemia.	Prognosis stratification application. Primarily employed for risk stratification and prognostic assessment in high-risk patients, particularly within the ATC classification system.	(132, 134, 135)
Exosomes	miRNA profile (e.g. miR-16-2-3p, miR-223-5p, miR-221-3p)	High potential. Specific miRNA combinations can effectively distinguish benign from malignant nodules, making them suitable for early screening of PTC.	Elevated levels of miR-221-3p correlate positively with tumor progression, and its overexpression constitutes a risk factor for PTC recurrence (HR = 1.41).	Early validation phase. Multiple biomarkers have demonstrated excellent diagnostic performance in retrospective studies, but lack standardised protocols and large-scale prospective validation.	(193, 194, 196, 197, 207, 208)
	ncRNA (e.g.lncRNA DOCK9-AS2, HOTAIR)	Focuses on reflecting the molecular functional state of tumors rather than direct early screening.	High. It reveals metastasis mechanisms such as tumour microenvironment communication and the dissemination of stem cell properties, and is a potential prognostic biomarker.	Basic research is transitioning towards translation. Mechanistic studies are advancing, but the development of clinically detectable biomarkers remains in its early stages.	(200, 201)

There is no single ‘universal’ biomarker, their practical value depends on specific clinical requirements. For example, ctDNA assays (such as ddPCR or NGS) are highly specific for detecting point mutations such as *BRAF^V600E^*, making them one of the most sensitive methods for monitoring MRD postoperatively. However, they are less sensitive for detecting fusion genes or in scenarios with low tumor burden. CTC analysis relies on complex enrichment and identification techniques (e.g. EpCAM-based immunocapture or size-based microfluidic chip sorting). Although it provides intact cells for functional studies, capture efficiency is low, particularly in early-stage patients, and standardized protocols are lacking. The main challenges associated with exosomes are their separation and purification (usually achieved via ultracentrifugation or commercial kits), and identifying tumor-specific RNA biomarkers (e.g. miR-146b-5p and miR-222-3p). Nevertheless, their stability in serum makes them a unique vehicle for long-term dynamic monitoring and microenvironmental signaling analysis. Therefore, marker selection should match specific clinical decision-making needs with corresponding technologies, rather than being a simple comparison of merits.

In the future, LB technology for TC should adopt a “scenario-driven, multi-modal integration” strategy. For example, in patients with high-risk PTC, ctDNA monitoring can be used to assess BRAF and other gene mutation statuses, while utilizing exosomal miRNA to evaluate metastatic propensity, enabling dynamic experimental monitoring. Furthermore, the integration of cutting-edge technologies such as microfluidics, single-cell sequencing, and artificial intelligence will undoubtedly propel the clinical management of TC into a new era characterized by greater precision and dynamic monitoring, ultimately guiding personalized therapeutic strategies and improving patient outcomes in TC.
